# Assessment of Pesticide Residue Content in Polish Agricultural Soils

**DOI:** 10.3390/molecules25030587

**Published:** 2020-01-29

**Authors:** Aleksandra Ukalska-Jaruga, Bożena Smreczak, Grzegorz Siebielec

**Affiliations:** Department of Soil Science Erosion and Land Protection, Institute of Soil Science and Plant Cultivation—State Research Institute, Czartoryskich 8, 24-100 Puławy, Poland; bozenas@iung.pulawy.pl (B.S.); gs@iung.pulawy.pl (G.S.)

**Keywords:** DDT/DDE/DDD, α-HCH, β-HCH, γ-HCH, atrazine, carbaryl, carbofuran, maneb, biodegradation, contaminants

## Abstract

Pesticides belong to a group of xenobiotics harmful to humans and wildlife, whose fate and activity depends on their susceptibility to degradation. Therefore, the monitoring of their residue level in agricultural soils is very important because it provides very valuable information on the actual level of soil contamination and environmental risk resulting from their application. The aim of this study was to evaluate contemporary concentrations of organochlorine (OCPs) and non-chlorinated pesticides (NCPs) in arable soils of Poland as an example of Central and Eastern European countries. The results were assessed in relation to Polish regulations, which are more restrictive compared to those of other European countries. The sampling area covered the territory of arable lands in Poland (216 sampling points). The distribution of sampling points aimed to reflect different geographical districts, conditions of agricultural production, and various soil properties. The collected soil samples were extracted with organic solvents in an accelerated solvent extractor (ASE 2000). The OCPs, including α-HCH, β-HCH, γ-HCH, and p,p’DDT, p,p’DDE, and p,p’DDD, were extracted with a hexane/acetone mixture (70:30 *v*/*v*) and determined by gas chromatography with an electron capture detector (GC-μECD). NCPs included atrazine, carbaryl, and carbofuran were extracted with a dichloromethane/acetone mixture (50:50 *v*/*v*), while maneb was extracted by intensive shaking the sample with acetone (1:1 *v*/*v*) and ethylenediamine-tertraacetic acid. The NCPs were identified by a dual mass- spectrometry (GC-MS/MS). The total content of individual OCPs ranged from 0.61 to 1031.64 µg kg^−1^, while the NCP concentrations were significantly lower, from 0.01 to 43.92 µg kg^−1^. DDTs were detected in all soils samples (p,p’DDD (23.60 µg kg^−1^) > p,p’DDT (18.23 µg kg^−1^) > p,p’DDE (4.06 µg kg^−1^), while HCHs were only in 4% of the analyzed samples (β-HCH (339.55 µg kg^−1^) > α-HCH (96.96 µg kg^−1^) > γ-HCH (3.04 µg kg^−1^)), but in higher values than DDTs. Among NCPs, higher concentration was observed for carbaryl (<0.01–28.07 µg kg^−1^) and atrazine (<0.01–15.85 µg kg^−1^), while the lower for carbofuran (<0.01–0.54 µg kg^−1^). Maneb was not detected in analyzed soils. Assessment of the level of soil pollution based on Polish regulations indicated that several percentages of the samples exceeded the criterion for OCPs, such as ∑3DDTs (14 samples; 6.5% of soils) and HCH congeners (α-HCH in one sample; 0.5% of soils), while NCP concentration, such as for atrazine, carbaryl and carbofuran were below the permissible levels or were not detected in the analyzed soils, e.g., maneb. The obtained results indicated that residues of the analyzed pesticides originate from historical agricultural deposition and potentially do not pose a direct threat to human and animal health. The behavior and persistence of pesticides in the soils depend on their properties. Significantly lower NCP concentration in the soils resulted from their lower hydrophobicity and higher susceptibility to leaching into the soil profile. OCPs are characterized by a high half-life time, which affect their significantly higher persistence in soils resulting from affinity to the soil organic phase.

## 1. Introduction

The use of pesticides significantly influences the intensification and efficiency of agriculture production [1,2]. Despite the high benefits on the economic value of crop production resulting from the application of plant protection products, their intensive and widespread use raises serious environmental concerns regarding the release of harmful substances into the environment [2,3,4]. More than 3000 different types of pesticides have been used in the European agricultural sector in the past 50 years [5]. The main problem is that a minor amount of pesticide compounds reaches their targets by the toxic effect to plant pathogens, while the rest constitute potential short- or long-term environment contaminants, with a wide range of possible negative impacts [3]. It has been recognized that less than 0.1% of applied pesticides protect crops, while the rest are directly deposited into the soil or subjected to off-site transport via wind or water [6]. Recent studies documented that current application rates exceed what is really necessary for effective crop protection [7].

The fate of pesticides in soil depends on many factors related to physical, chemical, and photochemical processes, as well as biological transformation [4,8]. Their behavior and persistence in soils are mainly determined by volatilization, plants uptake, leaching and runoff, sorption and binding by soil components, chemical degradation by hydrolysis, oxidation-reduction, photolysis, and degradation by soil micro-organisms [9,10,11]. These processes are related to environmental factors, including soil characteristics (texture), chemical properties (pH, organic matter and metal ions content), as well as climatic conditions [4,12,13]. The rate and direction of pesticide transformations ultimately determine their content and relative stability in soil [4,8]. The chemical properties of pesticides, as well as their coexistence with other chemical compounds that are often added to commercial products, i.e.,: surfactants, coagulants, decomposition inhibitors, buffer and synergic substances or adjuvants, may significantly modify the interactions of pesticides with soil components and affect their resistance to degradation [8,14].

Pesticide residues occurring in the soil constitute mainly toxic chemicals that adversely affect the environment [5]. Their excessive accumulation in the soil creates a serious risk of their transfer into the food chain or infiltration into groundwater [15]. Thus, soils contaminated by pesticide residues pose a serious problem, and were included in the Communication from the Commission to the Council and the European Parliament, the European Economic and Social Committee, and the Committee of the Regions: Toward a Thematic Strategy for Soil Protection [16]. Despite numerous documents and international actions, many countries do not have relevant soil monitoring programs that allow one to control the residue of toxic organic compounds. Moreover, existing regulations are not uniform for all countries [17]. Country-specific regulations include different permissible content or threshold limits of toxic organic compounds [2,17]. Most of the data are based on modeled values by predicting pesticide distribution in the soil environment [2]. Unfortunately, these data do not reflect the real and actual content or the bioavailability of toxic organic compounds in soil.

Agriculture is a very important economic sector in Europe. In Poland, rural areas cover over 95% of the total area of the country [18]. In 2018, arable land occupied over 14,000 thousand ha, which constituted less than half of Poland’s area [1,18]. According to the Polish Central Statistical Office, over the last 20 years, the use of pesticides significantly increased, from 8848 to 24,006 tons [18]. In 2019, the highest consumption level was recorded for herbicides and fungicides, 12,190 and 7737 tons, respectively, which accounted for 83% of all pesticides [18]. Nevertheless, to ensure the maintenance of the right quality of food production and environment protection, the actions to control the use of pesticides (analysis of active substances content, toxicology properties, half-life times and expected useful date) are conducted. Currently, in Poland [19,20,21,22,23,24] and in other countries of the European Union [25], very detailed and comprehensive legislation regulates and conditions the placing of plant protection products on the market. Additionally, the successive implementation of common agricultural policy assumptions resulted in the development of sustainable agriculture, which involves minimizing the consumption and degradation of land resources so that they can be used in the future to meet the basic needs of future generations. In 2013, Poland adopted the first national Action Plan to Reduce the Risks Related with the Use of Pesticides [26].

The environmental behavior of new-generation pesticides are under the requirements of the REACH directive (Registration, Evaluation, Authorisation and Restriction of Chemicals), but in many countries content of highly persistent and potentially toxic chlorinated pesticides such as: HCHs, DDTs, or drins (aldrine, deldrine, endrine) is still under control.

In 2016, the ordinance of the Polish Ministry of the Environment was amended, which indicates the toxic compounds causing risk to human health and soil whose content should be controlled [24]. According to these documents, the concentration of chlorinated-pesticide compounds (OCP) such as: sum of DDT (p,p’DDT, p,p’DDE, p,p’DDD), aldrin, dieldrin, endrin, α-HCH, β-HCH, γ-HCH and non-chlorinated pesticide compounds (NCP): carbaryl, carbofuran, atrazine, and maneb should be subjected to constant monitoring in soils. Although information on the pesticide residue content in Polish soils was included in the publications of several authors (Maliszewska-Kordybach et al. [27], Bojakowska and Gliwicz [28], Łozowicka et al. [29]) it is still unsatisfactory because a new toxic organic compounds group was included in the monitoring system. This manuscript provides broader analysis on the content of chlorinated- and non-chlorinated pesticide residues in arable Polish soils.

Our aim was to evaluate contemporary OCP and NCP concentrations in the arable soils of Poland as an example of Central and Eastern European countries. Results were assessed in relation to current Polish regulations.

## 2. Results and Discussion

### 2.1. Soil Physicochemical Properties

Generally, the basic physicochemical properties of soils exhibited relatively uniform values. The obtained data results (Table 1) had a similar to normal distribution after logarithmic transformation of the data. Most of the soils was characterized by high acidity, with a pH_KCl_ value ranging from 3.1 to 7.4 (mean = 5.0), maximum clay content (fr < 0.002 mm) of 47% with a mean at 5.2% (CoV = 111%), and silt content ranging from 2% to 83%. TOC concentrations were diversified from 3.6 to 38.4 g kg^−1^ (CoV = 44.6%), indicating the various degrees of supply inflow and accumulation of organic matter in agricultural soils. Similarly, TC and TN (3.6–39.4 and 0.4–3.6, respectively) with mean TC/TN ratio equaling 9.4 (CoV = 18.5%) indicated a varying degree of organic matter decomposition [30]. Results present the characteristic data for agricultural soils of Poland and other neighboring European countries [2,6,27,28,29,31].

### 2.2. OCP Soil Concentrations 

Statistical evaluation of OCPs concentrations in the soils is described in Table 2. Total OCP content ranged from 0.61 to 1037.59 µg kg^−1^. ∑3DDT compounds were detected in all soil samples (100%), and their concentrations were relatively uniform (CoV = 144%). The content of individual isomers was diversified, such as pp’DDT (0.12–202.68 µg kg^−1^), pp’DDE (<0.10–79.87 µg kg^−1^), and pp’DDD (<0.10–267.52 µg kg^−1^), which accounted for 35%, 8%, and 46% respectively, of total OCPs or ∑3DDT. Exceeding the permissible DDT content sum specified at the 120 µg kg^−1^ level [24] was only reported in 14 samples, which accounted for 6.5% of the analyzed soils. HCHs were detected only in 4% of the analyzed samples, but in higher concentrations than DDT. The sum of HCH compounds ranged from 0.98 to 1008.57 µg kg^−1^ with interquartile range 1.22-54.38 µg kg^−1^. The average contents of HCH congeners were as follow β-HCH (339.55 µg kg^−1^) > α-HCH (96.96 µg kg^−1^) > γ-HCH (3.04 µg kg^−1^). According to the results, HCH residue concentration exceeded the limit values included in Polish regulations in some soil samples (α-HCH = 25 µg kg^−1^, β-HCH = 10 µg kg^−1^, γ-HCH = 10 µg kg^−1^) [24]. OCP concentrations in agricultural soils in Poland are on similar or lower levels as in other European countries such as the Czech Republic (4.65–1022.3 µg kg^−1^ [31]), Germany (28.3–184.50 µg kg^−1^ [32]), Spain (0.08–448.0 µg kg^−1^ [33]), and the United Kingdom (0.1–100 µg kg^−1^ [33,34]). Furthermore, results are consistent with data obtained by Maliszewska-Kordybach et al. (0.35–453.2 µg kg^−1^) in a research conducted in 2005 [27]. However, the relationships between the level of pesticide residue content in various regions of the country (i.e., northern, southern, eastern and western parts of Poland, as well as individual voivodships, distance from cities, and soil type) were not observed. Therefore, differences in the compositional patterns of HCHs isomers or DDTs congeners, as well as the significant differences in their concentrations (ranging from less than 1 to over 100 μg/kg), predominantly resulted from the former intensity of pesticide application varied substantially among the agricultural regions in Poland. The degradation rate of these compounds was influenced by climate and soil environment conditions, sources of contamination, transport mechanisms and soil properties [35]. The high differences in OCP concentrations corresponded to data noted for German and Chinese agricultural soils [32,36]

The relatively high DDT residues in most monitored soils can be a consequence of permissible application of these compounds before 1970s, when the use of this pesticide was finally restricted [4,27,29,31]. It was estimated that DDT production reached approximately 0.7–0.8 million Mg in Europe. The extensive use of DDT resulted from the low costs of its manufacturing and high efficacy as an insecticide product. Despite Europe and the US banning the use of DDT, Asian countries and less economically developed countries in other continents [4,8,29,36,37,38] still use it. Therefore, DDTs can be transported with atmospheric particles to other countries and deposited in soils in temperate climate conditions. In our studies, the ratio of ∑3DDT and its isomers indicated the microbial transformation of the mother compound. The ratio of p,p’-DDE/p,p’DDT ranging from 0.10 to 14.83 can be used as an index to identify the fresh and/or aged application of DDT in a research area [8,35]. A value of <20 generally indicates an old deposition of these contaminants in the soil abandoned 15–20 years prior [35,37]. The confirmation of this thesis may be proportional (p,p’-DDD+p,p’-DDE)/∑3DDT, which in all sampling sites was >0.5 (from 0.93 to 2.46). Previous studies of Wang et al. [38] and Dirbaba et al. [8] showed that (p,p’DDE + p,p’DDD)/∑3DDT > 0.5 indicate historical application of DDTs products. Moreover, the ratio of (p,p’DDE + p,p’DDD)/∑3DDT > 0.5 indicates that aerobic transformation processes, which are typical for upland soils, are advantageous over anaerobic dechlorination, which is characteristic for sediments or waterlogged territories [8,35]. The high hydrophobicity of the DDT increases its ability to accumulate in the soil surface. Despite variations among sampling sites, this property might have contributed to a high concentration of ∑3DDT in the study area [9]. Moreover, a high proportion of p,p’-DDT and p,p’DDD can occur in the soils due to its low biodegradability and high octanol water partitioning coefficient, which influences the high potential for sorption by the organic matter fraction in soil, extending their resistance/persistence in soils [4].

Evaluation of HCH residue concentration indicated that the HCH sum was less widespread than the DDTs sum in the study area, although HCH had been more abundantly used in agricultural sector in Europe. Generally, HCHs have higher biodegradability and vapor pressure as compared to DDT that can lower their accumulation in soils. The highest percentage proportion of HCHs was contributed by β HCH, followed by α-HCH, and γ-HCH. Among all HCH congeners, β-HCH exhibited high persistence and very poor susceptibility to degradation in soils [8,27]. These properties might contribute to its content exceeding permissible limits in some regions in Poland. Dirbaba et al. [8] reported that γ-HCH can be biodegraded under aerobic condition to β-HCH under aerobic conditions in the natural environment. Thus, conversion of γ-HCH to β-HCH through microbial degradation might have contributed to the high concentrations of β-HCH in few samples from the study area [8]. The proportions of β-HCH/ΣHCHs above 0.5 indicated that β-HCH was the dominant HCH congener and majority of these compounds derive from historical deposition. According to the literature data [27,28], γ-HCH was the main component of commercial pesticide product lindane, which was extensively used in Poland. Moreover, atmospheric depositions also may have contributed to higher HCH concentration in soils, but Poland is the country with moderate annual deposition fluxes of γ-HCH [9,27]. High γ-HCH content in arable soils is dangerous due to its chemical and biological properties, generating a higher risk of leaching to groundwater and negative ecotoxicological effects, which directly threaten to maintain of properly soil quality. According to Jorfi et al. [4], risk assessment of agricultural soils contaminated with OCP residues indicated that these areas do not pose a serious threat to residents (via ingestion, dermal absorption, and inhalation) and vegetation.

### 2.3. NCP Soil Concentrations 

Statistical evaluation of NCP concentrations in soils is described in Table 2. The total content of NCPs ranged widely from value below LOD (<0.01 µg kg^−1^) to 43.92 µg kg^−1^. The interquartile range was narrow (0.3–1.02 µg kg^−1^) with an average value of 1.17 µg kg^−1^. The share of individual compounds in total NCP content was diversified (CoV = 343%). Higher concentration was observed for carbaryl (<0.01–28.07 µg kg^−1^) and atrazine (<0.01–15.85 µg kg^−1^) while lower for carbofuran (<0.01–0.54 µg kg^−1^). Maneb was not detected in any of the analyzed soils or its content was highly below the limit of detection. In all soils, the concentrations of NCP compounds did not exceed the permissible levels defined in the national regulation, namely for carbaryl: 200 µg kg^−1^, carbofuran: 200 µg kg^−1^, maneb: 200 µg kg^−1^, atrazine: 50 µg kg^−1^ [24]. The varying occurrence of individual NCPs compounds corresponds to their distinct properties such as persistence and sorption in soil and to their transformation routes.

Generally, the concentrations of individual NCPs in Polish soils are comparable with recorded results for other countries. Most studies concern atrazine which was highly detected in soils of Canada (1.01–52.49 µg kg^−1^ [39,40]), Croatia (5.0–25.0 µg kg^−1^ [41]), and China (1.38–86.34 [42]). Despite the theoretically short half-life time of atrazine (75 days, Table 3) Jablonowski et al. [43] found this compound and its degradation product (2-hydroxyatrazine) in soils 22 years after its application. These results suggest the high persistence of atrazine in soils and susceptibility to leaching from the top layer of the soil profile [44]. Atrazine was detected in groundwater in many countries, such as the United States [45], Brazil [46] and China [47], posing a high risk to living organisms. Yue et al. [48] indicated that the atrazine adsorption capacities of the soils markedly varied due to soil physicochemical properties, such as clay fraction and organic matter content. According to these authors, with the increase of atrazine concentration, adsorption quantity also increases. So, at a high concentration of atrazine, soil adsorption capacity presented a relatively simple and linear dependence trend, showing high capacity to retain this compound in the soil and increasing its stability [48].

The carbaryl concentration in Polish soils prevailed among all determined NCPs. Its amounts in soils result from the fact that carbaryl was used as a substitute for OCPs and it is one of the most commonly used insecticides still used in the United States [49]. Different studies established the presence of carbaryl and/or its hydrolyzed products in relatively high amounts in soils surface waters and rain runoff [50,51,52,53]. Carbaryl was found to adsorb more readily in acidic soil [37], which coincides with the obtained results for Polish soils. Recent research indicates that carbaryl sorption to the soil is rapid, with binding time ranging from 0.5 to 3 h. The fast accumulation of carbaryl in soils is caused by its high affinity to the mineral and organic fraction [49,50,53]. Both the mineral and the organic fraction are responsible for the stability of carbaryl in soil. Interactions with mineral soil components were clearly reported by Sheng et al. [54], who found that potassium (K)-saturated smectite clay (non-ionic, expandable, hydrophilic clay) is a better sorbent for carbaryl than soil organic matter. The authors proved that the distribution coefficient (K_o/w_) for carbaryl was five times higher in clay-rich soils (K_o/w_ = 235) than in soils with organic matter content exceeding 20 g kg^−1^ (K_o/w_ = 54.2). De Oliveira et al. [55] found that sorption of carbaryl is additionally controlled by the surface charge density and the occurrence of specific sorption sites. For example, the amount of carbaryl in soils was strongly dependent on the presence of specific exchangeable cations and followed the order: Ba ~ Cs ~ Ca > Mg ~ K > Na ~ Li [55]. Additionally, carbaryl sorption in soils depends on temperature. According to Singh et al. [54], temperature exerts an indirect influence on the adsorption process through its effect on carbamates solubility. An inverse relationship exists between the degree of adsorption and solubility. The increase of temperature raises the activation energy of reactive mineral or organic soil fractions to reduce sorption processes.

The concentration of carbofuran in analyzed Polish soils was very low, and this compound was only detected in four soil samples (about 2% of soils). This confirms the fast dissipation time of carbofuran in natural field conditions and its high susceptibility to degradation. These results are in agreement with the observations of Wang et al. [38], Otieno et al. [56], and Teerakun et al. [57], who stated that microbial degradation is an important decomposition pathway of carbofuran in neutral and acidic soils. Moreover, studies conducted by Lalah et al. [58] stated that carbofuran may adsorbed and metabolized in soil, giving a large number of indirect non-toxic metabolites. These processes are intensified through water, which provides a reaction medium for these transformations. Therefore, the noted concentration of carbofuran residue may be higher in topsoil during the wet season than during the dry season, since the compound easily dissolves and can be bound by the soil matrix in a shorter amount of time after application [56]. Furthermore, Singh et al. [40] reported the effect of water-miscible organic co-solvents that are applied into the soil with pesticides on carbofuran adsorption and movement in soils. The authors noted that some “accompanying hydrophobic compounds” may significantly prolong the stability or significantly affect the degradation of this compound after its deposition in soil.

Maneb, a polymeric complex of manganese (Mn) with ethylene bis dithiocarbamate [59] was not detected in Polish soils. Due to its chemical properties, it is a very unstable compound whose half-life time in the soil is estimated at 7 days at field conditions (Table 3). Therefore, its identification is particularly difficult due to its very rapid degradation [60]. Nevertheless, according to Vyas et al. [59], high organic matter content in soils or organic fertilization have a significant impact on increasing in its resistance and restricting the degradation potential of maneb. Moreover, clay content has significant influence on maneb retention, but this effect prevails only in soils with low organic matter content. Maneb is a broad-spectrum fungicide that is extensively applied against fungal pathogens [61]. Its extensive uses at higher doses and hazardous nature made it a global concern because the maneb molecule may transform into highly toxic compounds, such as etylenethiourea, mancozeb, or nabam, which are much more stable in soil (estimated half-life time, 30 days) and toxic (high Cramer class). 

### 2.4. Relationship between Soil Properties and Pesticide Residue Concentrations

**Figure 1:**
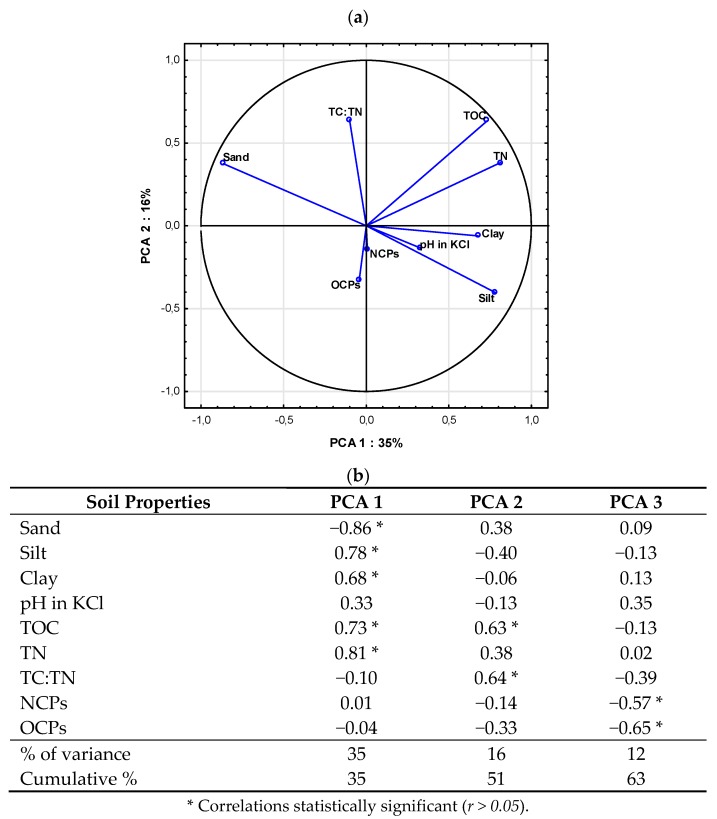
(**a**) Principal Component Analysis (PCA) ordination biplot (PCA 1 vs. PCA 2), eigenvectors of correlation matrix used to generate PCA components of soils contaminated by OCPs and NCPs pesticides, and seven measured soil variables for all data sets (*n* = 216). Arrows/lines in biplot represent variable loadings relative to each component. (**b**) Factor loading matrix; loading ≥ 0.5 in bold.

The relationship between soil physicochemical properties and pesticide residues concentrations is shown in Figure 1. All analyzed data are represented by three main components, explaining 63% of the total variance of the results (Figure 1b), whereas up to 51% of variance is explained by the first two factors (Figure 1a). The first PCA component (PCA 1), which accounted for 35% of variance, was significantly negatively correlated with sand (r = −0.86), and positively correlated with silt (r = 0.78) and clay (r = 0.68). The second PCA component (PCA 2), which represented merely 16% of data variance, was significantly positively correlated with TOC (r = 0.63) and TC/TN (r = 0.64), while the third component (PCA 3) was related with OCP (r = −0.65) and NCP (r = −0.57) chemical properties. This indicates that soil organic matter determines pesticide accumulation during aging to a greater extent than the content of organic matter and related parameters. Most of the vectors reach the circuit of the plot; therefore, all variables are well represented by the first two main components of the PCA coordinates. The distance (angle) between vectors confirmed a high mutual relation between soil physicochemical properties that only have a partial effect on pesticide behavior in soil.

Results indicate that pesticide residue formation and its persistence may also depend on other agricultural and environmental factors (e.g., concentration, application repeatability, microbiological activity, molecule chemical properties). Persistence of pesticides in soils was found to increase with increasing its concentration, whereas mineralization, and the formation of degradation products and bound residues, decrease at higher concentrations [62]. The formation of OCP and NCP residues may be mediated by activities of soil micro-organisms [9,36,63]. Microbial degradation of chemicals is controlled by the availability of the compound to the degrading microorganism. However, at high rates of pesticide application, high pesticides concentration may be inhibitory or toxic to the degrading micro-organisms [37]. Degradation is generally faster if compounds are suspended in the soil solution [12,62,63]. Pesticide physicochemical properties govern their behavior and biological activity in soil [63]. Molecular size, ionizability, volatility, water solubility, lipophilicity, and octanol water partitioning coefficient are key properties that dominate their half-life time in soil (Table 3) [9,36,63]. Nevertheless, half-life time depends on a number of external factors, including climate type and soil nature as well as number of pesticide applications and the type of associated compounds (solvents, surfactants, other agents). A clear understanding of the fate and behavior of pesticides following the consideration of the above issues is essential in the further development of pesticide control and risk management strategies in soils.

## 3. Materials and Methods

The research was conducted as a part of the national program: “Monitoring of the chemical properties of arable soils in Poland in 2015–2017 [64], including a sampling campaign carried out in 2015 on the whole Polish territory. Soil monitoring is an element of broader state monitoring of the environment. It was initiated in 1995 and it involves collecting georeferenced samples in permanent locations with a 5-year interval. This paper concentrates on the results of pesticides residue content in the soil samples collected across the entire country.

### 3.1. Soil Sampling

**Figure 2:**
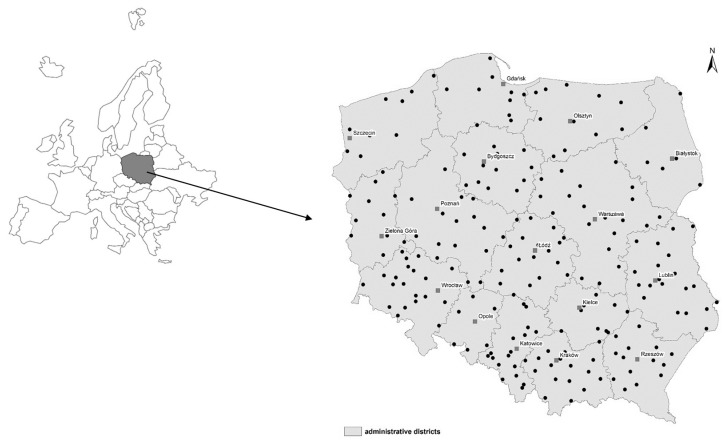
Sampling point map of research area (Poland).

The sampling area covered the arable lands in Poland (216 evenly distributed sampling points; Figure 2). It was aimed that the spatial distribution of the sampling points reflected different geographical districts, agricultural-production conditions, the magnitude of anthropogenic pressures, soil property variability, and representativeness of soil types and textures that are characteristic to Poland. The geographical location of sampling points was positioned on the basis of maps (1:25,000 scale) and precisely verified by the GPS technique. The soils were collected from the 0–20 cm depth, and an area of 100 m^2^, where the final sample was a mixture of 20 subsamples. Soil samples were air-dried (48 h at 20 °C), homogenized, sieved (2 mm sieve-mesh) and stored in the dark for further characterization.

### 3.2. Basic Soil Property Determination

The selected soil physicochemical properties included analysis of pH_KCl_, clay content (φ < 0.002 mm), total carbon content (TC), total organic carbon content (TOC) as well as total nitrogen content (TN) were measured. The pH was determined according to the PN-ISO 10,390 by potentiometric method and soil suspension in 1 mol l^−1^ KCl solution (1:2.5 m V^−1^). Clay content was analyzed according to the PN-R-04032 via the aerometric method, while TN and TC were determined by dry combustion in a Vario Macro cube CN elementar analyzer (Elementar Analysensysteme GmbH, Donaustraße 7, D-63452 Hanau-Germany). TOC content was determined according to the PN-ISO 14,235 by sulfochromic oxidation with titration of excess K_2_Cr_2_O_7_ with FeSO_4_(NH_4_)_2_SO_4_·6H_2_O.

### 3.3. OCP Concentration Determination

OCPs analysis comprised 3 congeners of HCH (α-HCH, β-HCH, and γ-HCH) and DDT (pp’DDT, pp’DDE, and pp’DDD)—Table 3. The analytical procedure were conducted according to the ISO10382 standard. Soil samples (10 g, grain size ≤ 0.10 mm) were mixed with diatomaceous earth (2 g) and extracted in accelerated solvent extractor (ASE200, Dionex Co., Sunnyvale, CA, USA) with a solvent mixture of hexane/acetone (70:30 *v*/*v*). Soil extracts were concentrated, and the solvent was exchanged to hexane. The concentrated extracts were passed through a glass column packed with glass wool, 2 g of deactivated alumina oxide (15% in MilliQ water), and 1 cm of anhydrous sodium sulfate; then it was eluted with 20 mL of petroleum ether. The solvent was exchanged to hexane following vacuum evaporation, and the extracts were concentrated to 1 mL. Determinations of DDTs and HCHs were performed using gas chromatography with microelectron capture detector (GC-µECD) system (Agilent 6890 Agilent Tech., Santa Clara, CA, USA). Quality control measures included analysis of certified reference material (CRM 847, ANAB Accredited Tested Laboratory), duplicate matrix samples, a solvent blank sample as well as surrogate and internal standards. As the surrogate standard, PCB 155 compound (2,2′,4,4′,6,6′-hexachlorobiphenyl, Dr. Ehrenstorfer GmbH, Augsburg, Germany) was used by soil spiking (10 g of soil was fortified by 10 µL of PCB 155 in a concentration of 20 µg mL^−1^), to estimate and control the matrix effects on the obtained results in the analyzed samples.

Additionally, PCB 207 (2,2′,3,3′,4,4′,5,6,6′-nonachlorobiphenyl; Dr. Ehrenstorfer GmbH, Augsburg, Germany) was used as the internal standard (added to the sample extract directly before injection), which allowed to control the instrument and injection parameters of GC-µECD.

OCPs soil extracts concentrated in *n*-hexane were analyzed in a single run using a gas chromatograph (Agilent 6890) equipped with a ^63^Ni microelectron capture detector (µECD) and Agilent 7683B Series autosampler (Agilent Technologies, Santa Clara, CA, USA). Samples were injected to a deactivated Agilent single-taper liner (p/n 5181-3316) in splitless mode at the injection-port temperature of 225 °C. DDTs were separated on a DB-5 capillary column (30 m × 320μm × 0.25 μm, Agilent Technologies; p/n 19091J-413). The remaining operating conditions of GC-µECD apparatus are presented in Table 4. OCP compounds were identified by comparing their retention times with chromatograms of standard solution mixtures and confirmed by GC MS/MS technique with multiple reaction monitoring mode (MRM).

The basic validation parameters were determined by analyzing the certified reference material (CRM 847, ANAB Accredited Tested Laboratory CRM 847) in 10 sample replicates and 2 repetitions of the injection of the same sample. Precision expressed as relative standard deviation (RSD), was in the range of 5–10%, and the recovery for individual compounds for certified reference material-CRM 847 was in the range of 71–82% for the DDTs, and 67–89% for the HCHs. The limit of detection (LoD) for DDT and HCH compounds was at the 0.10 μg·kg^−1^ level. The detection limit value was adopted as the minimal value of the content of the measured OCPs compounds. More detailed analysis methodology was described by Maliszewska–Kordybach et al. [27].

### 3.4. NCP Concentration Determination

NCPs analysis included determination of atrazine, carbaryl, carbofuran, and maneb (Table 3) in two separated analytical steps. The analytical procedures were based on the extraction of dried ground soil samples (10g, grain size ≤0.10 mm) mixed with 2 g of diatomaceous earth. The atrazine carbaryl and carbofuran were extracted with a mixture of dichloromethane and acetone (50:50 *v*/*v*) in an ASE200 (ASE200, Dionex Co., Sunnyvale, CA, USA) apparatus. Clean-up of the concentrated extract was performed using dichloromethane:ethyl acetate elution (50:50 *v*/*v*) in a glass column packed with glass wool, 2 g of deactivated alumina oxide (15% in MilliQ water), and 1 cm of anhydrous sodium sulfate. The solvent was exchanged to ethyl acetate following vacuum evaporation, and the extracts were concentrated to 1 mL.

Maneb was extracted according to methodology described in the Handbook of Pesticides-Methods of Pesticides Residue Analysis (CRC Press Boca Raton, FL, USA, Leo M.L. Nollet, Hamir S. Rothore) by intensive shaking of the soil sample with acetone (1:1 *v*/*v*) in an ultrasonic bath. To efficiently dissolve maneb in acetone, chelating agents such as ethylenediamine-tertraacetic acid (EDTA), were additionally used to complex the maneb molecules and strip away the manganese bivalent agent (derivatization processes). The released ethylene-bis-dithiocarbamate (EBDTC) anions were stabilized by the addition of ascorbic acid to prevent their decomposition processes. The obtained extracts were purified in the column with activated carbon (to remove particles of organic matter), and then the extracts were concentrated by evaporation to 1 mL. Samples were analyzed on GC MS/MS (full SCAN scan ion monitoring mode) up to 5 days after their extraction due to the possibility of extraction product decomposition. Maneb was identified and quantified by comparing the retention time peaks of the standard solutions. The efficiency of the derivatization process was determined with analyses of the 10 samples of the prepared standard solutions (PESTANAL^®^, analytical standard, no. 45554-250MG); on average 87% with RSD at 4%. The recovery of the applied method was determined by maneb addition to the soil at level of 400 µg kg^−1^ which corresponds to a double limit value of the permissible maneb content in Polish agricultural soils (JoL 2016, item 1395). The spiked soil sample was homogenized (mixing, grinding), divided into 10 sub-samples and subjected to the extraction procedure. Method recovery was noted at the level of 78%, while method repeatability at 6%. Indeterminate and random errors were not detected in the developed methods. The correctness of the method was confirmed by HPLC (absorbance wavelength at 232 nm; acetonitryl/water at 10:90 *v*/*v*; flow rate 1 mL/min).

Similarly to OCP analysis, we used the PCB 155 compound (2,2′,4,4′,6,6′-hexachlorobiphenyl, Dr. Ehrenstorfer GmbH, Augsburg, Germany) as surrogate standard by soil spiking (10 g of soil was fortified by 10 µL PCB 155 in a concentration of 20 µg mL^−1^), to estimate and control the matrix effects in the analyzed samples. Additionally, PCB 207 (2,2′,3,3′,4,4′,5,6,6′-nonachlorobiphenyl, Dr. Ehrenstorfer GmbH, Augsburg, Germany) was used as internal standard (added to the sample extract directly before injection) that allowed one to control instrument and injection GC MS/MS parameters.

NCP concentrations in the extracted samples were determined by gas chromatography triple mass spectrometry (GC-MS/MS; Agilent 7890B GC system; Agilent Tech., Santa Clara, CA, USA), equipped with an Agilent 7000C detector and Agilent 7693 Autosampler. Table 4 displays the GC, backflush, and MS/MS method parameters. GC was configured with a Multimode Inlet (MMI) equipped with a 4 mm ultra-inert liner, splitless, single taper, glass wool liner (p/n 5190-2293). From the inlet, 2 HP-5ms UI columns (0.7 m × 150 μm, p/n 160-2625-5; and 30 m × 250 μm × 0.25 μm, p/n 19091S-431 UI, Agilent Technologies) were coupled to each other through a purged ultimate union for the use of midcolumn/post run backflushing. Sample analyses of atrazine, carbaryl, and carbofuran were performed in multiple reactor monitoring (MRM) mode with individual diagnostic ions, while maneb was analyzed in selected ion monitoring (SIM) mode.

The precision of the method expressed as a relative standard deviation (RSD) was in the range of 3–10% and the recovery for individual compounds for certified reference material BNA in the soils—PT (SQC003, Supelco) was within 86–90%. The limit of detection (LoD) for individual NCPs compounds was at the 0.01 µg kg^−1^ level. The detection limit value was adopted as the minimal value of the content of measured NCPs compounds.

### 3.5. Statistics

Received data were analyzed by basic statistical parameters, namely, minimum (Min), maximum (Max), median (Me), lower (LQ), upper quartile (UQ), standard deviation (SD), coefficient of variation (CoV), kurtosis and skewness. The normal distribution of the results was checked by chi-squared test. Principal component analysis (PCA) was performed to provide an overview of the relation pattern between OCP and NCP concentrations in soils and soil properties. PCA was used to evaluate the inter-relationships in measured variables in order to identify the essential structure of their correlations.

## 4. Conclusions

Contamination of arable soils in Poland representing Central and Eastern Europe, by organochlorine and nonchlorinated pesticide residues was generally low, comparable to contamination levels observed in the rural areas of other European countries. Assessment of the level of soil contamination based on Polish regulations [24] indicated that only some of the sample percentage exceeded the criterion for OCP content, such as the DDT sum (14 samples, 6.5% of soils) and HCH congeners (α-HCH in one sample, 0.5% of soils). While NCP concentration such as atrazine, carbaryl, and carbofuran were below the permissible levels, these compounds were not detected in the analyzed soils, e.g., maneb. The obtained results pointed out that residues of the analyzed pesticides rather originate from historical deposition and do not pose a potential direct threat to human and animal health. The behavior and persistence of pesticides in soils depend on their properties. Significantly lower NCP concentration in soils results from their lower hydrophobicity and higher susceptibility to leaching into the soil profile. OCPs are characterized by high half-life time that affects their significantly higher persistence in soils, resulting from affinity to the organic phase of soils.

## Figures and Tables

**Table 1:** Physicochemical soil properties (*n* = 216).

Soil Properties	Min	Max.	Me	CoV	LQ	UQ
Clay (%)	0.0	47.0	3.0	112	2.0	6.0
Silt (%)	2.0	83.0	28.0	62	19.0	56.0
Sand (%)	11	97	66	43	35	78
pH in KCl	3.1	7.4	5.0	21	4.2	5.9
TOC (g kg^−1^)	3.6	38.4	9.8	45	8.3	3.1
TN (g kg^−1^)	0.4	3.6	1.1	42	0.1	0.1
TC/TN	4.03	15.6	9.4	18	8.2	10.1

TOC, total organic carbon; TN, total nitrogen; Min, minimum; Max, maximum; Me, median; CoV, coefficient of variance; LQ, lower quartil; UQ, upper quartil.

**Table 2:** Results of OCPs and NCPs concentrations in Polish agricultural soils (*n* = 216).

	Min	Max	Aver	Me	LQ	UQ	SD	CoV	Kurtosis	Skewness	No. and % of Samples with Detected Pesticide Compound	Acceptable Limits (JoL 2016 Item 1395)
	Organochlorine Pesticides (OCPs)											
pp’DDT	0.12	202.68	18.23	8.54	2.47	22.50	26.91	148	16	4	216 (100%)	-
pp’DDE	<0.1	79.87	4.06	1.53	0.56	4.06	8.65	213	41	3	211 (98%)	-
pp’DDD	<0.1	267.52	23.60	9.58	4.25	25.33	39.56	168	18	6	211 (98%)	-
∑ DDT	0.61	484.64	44.60	24.73	8.78	53.47	64.35	144	17	4	216 (100%)	120
α-HCH	<0.1	192.64	96.96	96.96	49.12	144.80	135.32	140	−3	1	4 (2%)	25
β-HCH	<0.1	1008.57	339.55	8.57	5.04	508.57	579.40	171	−1	2	6 (3%)	10
γ-HCH	<0.1	7.27	3.04	1.53	1.17	4.79	2.78	92	−1	1	6 (3%)	10
∑ HCH	0.98	1008.57	152.78	3.70	1.22	54.38	352.31	231	7	6	8 (4%)	-
∑ OCPs	4.03	1037.59	61.70	34.63	15.86	66.46	97.62	158	49	3	216 (100%)	-
	Nonchlorinated Pesticides (NCPs)											
Atrazine	<0.01	15.85	0.63	0.38	0.28	0.56	1.27	201	122	10	173 (80%)	50
Carbaryl	<0.01	28.07	2.11	0.77	0.38	1.38	4.59	3	24	5	45 (20%)	200
Carbofuran	<0.01	0.54	0.45	0.46	0.40	0.51	0.07	17	−3	0	4 (2%)	200
Maneb	n.d	n.d	n.d	n.d	n.d	n.d	n.d	n.d	n.d	n.d	0 (0%)	200
∑ NCPs	<0.01	43.92	1.17	0.46	0.30	1.02	3.58	308	118	10	176 (82%)	-

Note: all values expressed in µg kg^−1^, except CoV, expressed in %; value below detection limit was adopted as minimum value.

**Table 3:** Chemical and physical properties nonchlorinated-pesticide compounds (NCPs) and chlorinated-pesticide compounds (OCPs) *.

	OCPs						NCPs			
	pp’DDT	pp’DDE	pp’DDD	α-HCH	β-HCH	γ-HCH	Atrazine	Carbaryl	Carbofuran	Maneb
Molecule structure	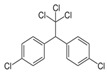	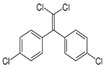	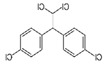	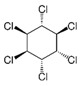	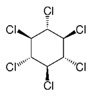	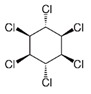	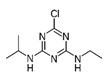	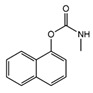	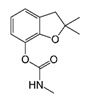	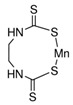
Pesticide type	Insecticide	Insecticide	Insecticide	Insecticide	Insecticide	Insecticide	Herbicide	Insecticide	Insecticide	Fungicide
Molecular weight (g mol^−1^)	354.49	318.02	320.04	290.82	290.82	290.82	215.68	201.22	221.26	265.30
Water solublility (mg l^−1^)	0.006	0.12	0.09	2.0	2.41	8.52	35	9.1	322	178
log K_o/w_	6.91	6.51	6.02	3.82	3.57	3.50	2.7	2.36	1.8	−0.45
Vapor pressure (mPa)	0.025	-	0.18	5.99	0.029	4.40	0.039	0.042	0.08	0.014
Soil degradation, DT 50 (days)	6200	5000	1000	175	10	980	75	16	29	7
Bioconcentration factor (l kg^−1^)	3173	1800	-	20	527	1300	4.3	44	12	Low risk
Threshold of toxicological concern (Cramer class)	High (class III)	High (class III)	High (class III)	High (class III)	High (class III)	High (class III)	High (class III)	High (class III)	High (class III)	High (class III)

Log K_o/w_—logarithm of the octanol-water partitioning coefficient. * Data from PubChem Open chemistry database and PPDB: Pesticide Properties DataBase University of Hertforddhire.

**Table 4:** Gas chromatography with an electron capture detector (GC-µECD) and dual mass- spectrometry (GC-MS/MS) method conditions.

Parameter	GC-µECD	GC-MS/MS
Injection mode	Splitless	Hot-splitless; MMI injection mode
Injection volume	2 μL	2 μL
Inlet temperature	225 °C	280 °C
Carrier gas	He constant flow 2.00 mL/min	He, constant flow 1.00 mL min^−1^ (column 2 = 1.20 mL min^−1^)
Detector temperatutre	325 °C	-
Makeup gas	N_2_, constant flow 40 mL min^−1^	-
Oven program	50 °C for 1 min, 30 °C/min to 180 °C, 180 °C, for 1 min, 3 °C/min to 205 °C, 205 °C for 4 min, 20 °C/min to 290 °C, 290 °C for 7 min	70 °C for 2 min 25 °C/min to 150 °C for 0 min; 3 °C/min to 200 °C for 0 min; 8 °C/min to 280 °C for 10 min hold time
MS transfer line temperature	-	280 °C
Backflush settings	-	5 min during post-run/310 °C
Aux EPC pressure	-	~50 psi
Inlet pressure	-	~2 psi
Column pressure	-	~3 psi
Electron energy	-	70 eV
MS1 and MS2 resolution	-	Wide
Collision cell	-	1.5 mL min^−1^ N_2_ and 2.25 mL min^−1^ He
Source temperature	-	300 °C
Quad temperatures	-	150 °C

## References

[1] Mrówczyńska-Kamińska A. (2008). The Importance of Agriculture in the Polish National Economy: Macroeconomic and Regional Analysis. Problems of World Agriculture. Sci. J. Warsaw Univ. Life Sci..

[2] Silva V., Mol H., Zomer P., Tienstra M., Ritsema C.J., Geissena V. (2019). Pesticide Residues in European Agricultural Soils—A hidden Reality Unfolded. Sci. Total. Environ..

[3] Hvězdová M., Kosubová P., Košíková M., Scherr K., Šimek Z., Brodský L., Šudoma M., Škulcová L., Sáňka M., Svobodová M. (2018). Currently and Recently Used Pesticides in Central European Arable Soils. Sci. Total. Environ..

[4] Jorfi F., Atashi Z., Akhbarizadeh R., Khorasgani Z., Ahmadi M. (2019). Distribution and Health Risk Assessment of Organochlorine Pesticides in Agricultural Soils of the Aghili Plain, Southwest Iran. Environ. Earth Sci..

[5] FAO (2017). ITPS Global Assessment of the Impact of Plant Protection Products on Soil Functions and Soil Ecosystems.

[6] Stolte J., Tesfai M., Øygarden L., Kværnø S., Keizer J., Verheijen F., Panagos P., Ballabio C., Hesse R. (2016). Soil Threats in Europe: Status, Methods, Drivers and Effects on Ecosystem Services.

[7] Lechenet M., Dessaint F., Py G., Makowski D., Munier-Jolain N. (2017). Reducing Pesticide Use While Preserving Crop Productivity and Profitability on Arable Farms. Nat. Plants.

[8] Dirbaba N.B., Li S., Wu H., Yan X., Wang J. (2018). Organochlorine Pesticides, Polybrominated Diphenyl Ethers and Polychlorinated Biphenyls in Surficial Sediments of the Awash River Basin, Ethiopia. PLoS ONE.

[9] Cycoń M., Piotrowska-Seget Z. (2006). Transformations of Pesticides in Soil Environment—A review. Pestycydy.

[10] Ehlers G., Loibner A. (2006). Linking Organic Pollutant (bio)availability with Geosorbent Properties and Biomimetic Methodology: A Review of Geosorbent Characterization and (bio)availability Prediction. Environ. Pollut..

[11] Nieder R., Benbi D.K., Reichl Z.X. (2018). Soil-Borne Particles and Their Impact on Environment and Human Health. Soil Components and Human Health. Springer Neth..

[12] Yang H., Wu X., Zhou L., Yang Z. (2005). Effect of Dissolved Organic Matter on Chlorotoluron sorption and desorption in soils. Pedosphere.

[13] Pu X., Cutright T. (2006). Sorption–Desorption Behavior of PCP on Soil Organic Matter and Clay Minerals. Chemosphere.

[14] Laha S., Tansel B., Ussawarujikulchai A. (2009). Surfactant–Soil Interactions during Surfactantamended Remediation of Contaminated Soils by Hydrophobic Organic Compounds: A review. J. Environ. Manag..

[15] Babut M., Arts G.H., Caracciolo A.B., Carluer N., Domange N., Friberg N. (2013). Pesticide risk assessment and management in a globally changing world—report from a European interdisciplinary workshop. Environ. Sci. Pollut. Res. Int..

[B16-molecules-25-00587] 16.COM 231 Final Communication from the Commission to the Council and the European Parliament, the European Economic and Social Committee and the Committee of the Regions: Towards a thematic strategy for soil protection; COM(2006)231 final Brussels, Belgium 2006.

[17] Carlon C. (2007). Derivation Methods of Soil Screening Values in Europe. A Review and Evaluation of National Procedures towards Harmonization.

[18] Statistical Yearbook of Agriculture **2018** (in Polish). https://stat.gov.pl/obszary-tematyczne/roczniki-statystyczne/roczniki-statystyczne/rocznik-statystyczny-rolnictwa-2018,6,12.html.

[19] JoL 2013 item 1686 Regulation of Minister of Agriculture and Rural Development from 13th December, **2013** on Certifying of Equipment’s Technical Efficiency. http://prawo.sejm.gov.pl/isap.nsf/DocDetails.xsp?id=WDU20130001686.

[20] JoL 2013 item 1742 Regulation of Minister of Agriculture and Rural Development from 18th December, **2013** on Requirements Regulating Equipment’s Technical Efficiency. http://prawo.sejm.gov.pl/isap.nsf/DocDetails.xsp?id=WDU20130001742.

[21] JoL 2013 item 554 Regulation of Minister of Agriculture and Rural Development from 8th May, **2013** on pesticides’ trainings. http://prawo.sejm.gov.pl/isap.nsf/DocDetails.xsp?id=WDU20130000554.

[22] JoL 2013 item 625 Regulation of Minister of Agriculture and Rural Development from 22nd May, **2013** on ways of pesticides’ use and storage. http://prawo.sejm.gov.pl/isap.nsf/DocDetails.xsp?id=WDU20130000625.

[23] JoL 2014 item 516 Regulation of Minister of Agriculture and Rural Development from 31st March, **2014** on terms of use of pesticides. http://prawo.sejm.gov.pl/isap.nsf/DocDetails.xsp?id=WDU20140000516.

[24] JoL 2016 item 1395 Regulation of Minister of Environmental Protection from 1th September, **2016** on assessment of soil surface pollution. http://prawo.sejm.gov.pl/isap.nsf/DocDetails.xsp?id=WDU20160001395.

[25] Commission Directive (EU) 2019/782 of 15 May 2019. https://eur-lex.europa.eu/legal-content/EN/TXT/PDF/?uri=CELEX:32019L0782&from=EN.

[26] Report on the Implementation of Directive 2009/128/EC on the Sustainable Use of Pesticides (2017/2284(INI)) 2019. http://www.europarl.europa.eu/doceo/document/A-8-2019-0045_EN.html.

[27] Maliszewska-Kordybach B., Smreczak B., Klimkowicz-Pawlas A. (2014). Evaluation of the Status of Contamination of Arable Soils in Poland with DDT and HCH Residues; National and Regional Scales. Pol. J. Environ. Stud..

[28] Bojakowska I., Gliwicz T. (2005). Chlorinated Pesticides and Polychlorinated biphenyls in river sediments of Poland. Polish Geol. Rev..

[29] Łozowicka B., Kaczyński P., Wolejko E., Piekutin J., Sagitov A., Tolwubayev K., Isenova G., Abzeitova E. (2016). Evaluation of Organochlorine Pesticide Residues in Soil and Plants from East Europe and Central Asia. Desalin. Water. Treat..

[30] Tan K.H. (2014). Humic Matter in Soil and Environment. Principles and Controversies, Boca Raton, FL 33487-2742.

[31] Holoubek I., Dušek L., Sáňka M., Hofman J., Čupr P., Jarkovskỳ J., Zbíral J., Klánová J. (2009). Soil Burdens of Persistent Organic Pollutants—Their Levels, Fate and Risk. Part I. Variation of Concentration Ranges According to Different Soil Uses and Locations. Environ. Pollut..

[32] Manz M., Wenzel K.D., Dietze U., Schüürman G. (2001). Persistent Organic Pollutants in Agricultural Soils of Germany. Sci. Total Environ..

[33] Cabrerizo A., Dachs J., Jones K.C., Barcel D. (2011). Soil-Air Exchange Controls on Background Atmospheric Concentrations of Organochlorine Pesticides. Atmos. Chem. Phys..

[34] Meijer S.N., Halsall C.J., Harner T., Peters A.J., Ockenden W.A., Johnston A.E., Jones K.C. (2001). Organochlorine Pesticide Residues in Archived UK Soil. Environ. Sci. Technol..

[35] Maliszewska-Kordybach B., Smreczak B., Klimkowicz-Pawlas A. (2013). The Levels and Composition of Persistent Organic Pollutants in Alluvial Agriculture Soils Affected by Flooding. Environ. Monit. Assess..

[36] Gao J., Zhou H., Pan G., Wang J., Chen B. (2013). Factors Influencing the Persistence of Organochlorine Pesticides in Surface Soil from the Region Around the Hongze Lake, China. Sci. Total Environ..

[37] Rajendran R.B., Imagawa T., Tao H., Ramesh R. (2005). Distribution of PCBs, HCHs and DDTs and their Ecotoxicological Impacs in Bay of Bengal, India. Environ. Int..

[38] Wang W., Junhong B., Guangliang Z., Xin W., Jia J., Baoshan C., Xinhui L. (2017). Depth-Distribution, Possible Sources, and Toxic Risk Assessment of Organochlorine Pesticides (OCP s) in Different River Sediment Cores Affected by Urbanization and Reclamation in a Chinese Delta. Environ. Pollut..

[39] Messing P.G., Farenhorst A., Waite D.T., McQueen D.A.R., Sproull J.F., Humphries D.A., Thompson L.L. (2011). Predicting Wetland Contamination from Atmospheric Deposition Measurements of Pesticides in the Canadian Prairie Pothole Region. Atmos. Environ..

[40] Singh B., Farenhorst A., Gaultier J., Pennock D., Degenhardt D., McQueen R. (2014). Soil Characteristics and Herbicide Sorption Coefficients in 140 Soil Profiles of two Irregular Undulating to Hummocky Terrains of Western Canada. Geoderma.

[41] Stipičević S., Galzina N., Udiković-Kolić N., Jurina T., Mendaš G., Dvoršćak M., Petrić I., Barić K., Drevenkar V. (2015). Distribution of Terbuthylazine and Atrazine Residues in Crop-Cultivated soil: The Effect of Herbicide Application Rate on Herbicide Persistence. Geoderma.

[42] Chung N., Alexander M. (2002). Effect of Soil Properties on Bioavailability and Extractability of Phenanthrene and Atrazine Sequestered in Soil. Chemosphere.

[43] Jablonowski N.D., Köppchen S., Hofmann D., Schäffer A., Burauel P. (2009). Persistence of 14C-labeled Atrazine and its Residues in a Field Lysimeter Soil after 22 years. Environ. Pollut..

[44] Martinsa E., Melob V., Bohonea J., Abatea G. (2018). Sorption and Desorption of Atrazine on Soils: The Effect of Different Soil Fractions. Geoderma.

[45] Toccalino P.L., Gilliom R.J., Lindsey B.D., Rupert M.G. (2014). Pesticides in Groundwater of the United States: Decadal-Scale Changes, 1993–2011. Groundwater.

[46] Vonberg D., Vanderborght J., Cremer N., Pütz T., Herbst M., Vereecken H. (2014). 20 years of Long-Term Atrazine Monitoring in a Shallow Aquifer in Western Germany. Water Res..

[47] Montagner C.C., Vidal C., Acayaba R.D., Jardim W.F., Jardim I.C.S.F., Umbuzeiro G. (2014). Trace Analysis of Pesticides and An Assessment of their Occurrence in Surface and Drinking Waters from the State of São Paulo (Brazil). Anal. Methods.

[48] Yue L., Ge C.J., Feng D., Yu G., Deng H., Fu B. (2017). Adsorption–Desorption Behavior of Atrazine on Agricultural Soils in China. J. Environ. Sci..

[49] Siampiringue M., Chahboune R., Wong-Wah-Chung P., Sarakha M. (2019). Carbaryl Photochemical Degradation on Soil Model Surfaces. Soil Syst..

[50] Demirbas A. (1998). Spectrophotometric Determination of Carbaryl Pesticide and its Product in Soil and Strawberry Samples. Sci. Total Environ..

[51] Martin J.D., Crawford C.G., Larson S.J. (2003). Pesticides in Streams—Preliminary Results from Cycle I of the National Water Quality Assessment Program (NAWQA).

[52] Walters J., Goh K.S., Li L., Feng H., Hernandez J., White J.J. (2003). Environmental Monitoring of Carbaryl Applied in Urban Areas to Control the Glassy-Winged Sharpshooter in California. Environ. Monit. Assess..

[53] Kinyunzu J.M. (2015). Residues Concentrations of Carbaryl Pesticide in Soil and Tomatoes from Hippo, Kingfisher and Harnekop Green House Farms in Thika and Naivasha, Kenya. Ph.D. Thesis.

[54] Singh R.P., Singh S., Srivastava G. (2011). Adsorption Thermodynamics of Carbaryl onto Four Texturally Different Indian Soils. Adsorp. Sci. Technol..

[55] De Oliveira M.F., Johnston C.T., Premachandra G.S., Teppen B.J., Li H., Laird D.A., Zhu D., Boyd S.A. (2005). Spectroscopic Study of Carbaryl Sorption on Smectite from Aqueous Suspension. Environ. Sci. Technol..

[56] Otieno P., Lalah O., Virani M., Jondiko I., Werner Schramm K. (2010). Soil and Water Contamination with Carbofuran Residues in Agricultural Farmlands in Kenya Following the Application of the Technical Formulation Furadan. J. Environ. Sci. Health. Part B.

[57] Teerakun M., Reungsang A., Virojanakud W. (2004). Phytoremediation of Carbofuran in Soil. Environ. Hazard. Manag..

[58] Lalah J.O., Kaigwara P.N., Getenga Z., Mghenyi J.M., Wandiga S.O. (2001). The Major Environmental Factors that Influence Rapid Disappearance of Pesticides from Tropical Soils in Kenya. Toxicol. Environ. Chem..

[59] Vyas B., Kumar Singh A., Singh Cameotra S. (2015). Sorption Behaviour of Maneb in the Agriculture Soils and its Correlation with Soil Properties. Int. J. Eng. Sci..

[60] Grimalt S., Dehouck P. (2016). Review of Analytical Methods for the Determination of Pesticideresidues in Grapes. J. Chromatogr. A.

[61] Bhushan C., Bhardwaj A., Misra S. (2013). State of Pesticide Regulations in India.

[62] Gan J., Koskinen W.C., Becker R.L., Buhler D.D. (1995). Effect of Concentration on Persistence of Alochlor in soil. J. Environ. Qual..

[63] Gevao B., Semple K., Jones K. (2000). Bound Pesticide Residues in Soils: A Review. Environ. Pollut..

[64] Siebielec G., Smreczak B., Klimkowicz-Pawlas A., Kowalik M., Kaczyński R., Koza P., Ukalska-Jaruga A., Łysiak M., Wójtowicz U., Poręba L. (2017). Report for the III Stage of the Monitoring of the Chemical Properties of Arable Soils in Poland in Years 2015–2017. Chief Inspectorate of Environmental Protection, IUNG-PIB, 2017. http://www.gios.gov.pl/images/dokumenty/pms/monitoring_jakosci_gleb/Raport_MChG_etap3.pdf.

